# Inequality of opportunity in health: evidence from Chile

**DOI:** 10.11606/S1518-8787.2017051007034

**Published:** 2017-11-22

**Authors:** Kevin Gallardo, Liseth Varas, Mauricio Gallardo

**Affiliations:** I Universidad Católica del Norte. Escuela de Ciencias Empresariales. Coquimbo, Chile

**Keywords:** Health Status, Epidemiologic Factors, Socioeconomic Factors, Health Inequalities

## Abstract

**OBJECTIVE:**

To assess the possible presence of inequality of opportunities in the health status of Chileans, according to sociodemographic circumstances.

**METHODS:**

Self-rated health data were used from the Chilean National Health Survey of 2010 to test the hypothesis of strong and weak equality of opportunities in the health status of the Chilean population. These hypotheses were tested using nonparametric techniques and second-order stochastic dominance criteria.

**RESULTS:**

Robust empirical evidence was obtained, which indicate that the education level of the mother, household socioeconomic status, sex, zone, and region of residence determine opportunities to achieve good health in Chile.

**CONCLUSIONS:**

Better health status was identified for Chilean adults whenever their mothers had a higher education level, their household income was higher, they were men, or lived in urban areas. The region of residence also affects opportunities to achieve good health in Chile.

## INTRODUCTION

Over the past three decades, Chile has achieved a significant increase in *per capita* income in relation to other Latin American countries. Nevertheless, it continues to be a very unequal country, with the highest Gini index on disposable income among the members of the Organization for Economic Cooperation and Development (OECD). This social problem has been a major focus of the political and academic debate in Chile, especially in terms of inequality related to income and education. Less attention has been devoted to health inequality. Research in this area of welfare is still scarce. The international arena, for its part, has long recognized the importance of acting on the social determinants of health to address the existing inequities^26^.

Among the few published research works on health inequalities in Chile, some have focused on specific regions and institutions of the country. For the adult population of the city of Concepción, Vidal et al.^25^ have found differences in the average outcomes of health-promoting lifestyle, according to sex, age range, income range, and education level. Meanwhile, Sanchez and Albala^20^ have conducted a measurement exercise on territorial inequality in mortality for the communes of the Santiago Metropolitan Region. They have found that mortality in these communes is correlated with income. In another study, Castro et al.^5^ have used prospective data from 14 hospitals to show that the average incidence of heart failure in hospitalized patients in Chile depends on the education level. There are also two studies on health inequality in Chile with national coverage. One of them^23^ assesses the differences in self-rated health (SRH) in relation to income levels, using data from the Chilean National Socioeconomic Characterization Survey (CASEN). This relationship is explored by implementing a multilevel logistic regression analysis, in which SRH is reduced to two categories and income levels are taken as the explanatory variables, while controlling for demographic factors. This study has found that the probability of reporting poor SRH increases as income lowers. Contreras et al.^7^ have also conducted a study using CASEN data; this is the only study for Chile in which the equality of opportunity approach has been applied to health outcomes. However, their research has focused on two particular categorical outcomes: (i) access to drinking water and sanitation and (ii) access to adequate child nutrition. Moreover, they have measured inequalities of opportunity considering only one circumstance: the region of residence.

This research differs from previous studies on health inequalities in Chile in several aspects. First, it covers the entire country using data from the Chilean National Health Survey (ENS), instead of the CASEN. Second, instead of assessing a particular aspect of health, this study uses general health status as the focal variable, measuring it in terms of SRH in five categories. As is known^1^, the SRH has been increasingly used internationally in comparative health studies among population groups and in research on social inequalities in health. Moreover, this study follows the inequality of opportunity^18^ (IO) approach, rather than the traditional approach of inequality of outcomes. The difference between these two methodological perspectives is significant. In the traditional approach, the social loss of inequality is assessed under some social welfare function^4^ while taking into account only the effective outcomes obtained by persons (e.g., monetary income, education level, or health conditions). The IO approach^18^ instead establishes a conceptual and normative distinction in relation to the elements that determine the inequality of outcomes. Inequalities arising from individual effort and personal decisions are not considered unfair and morally reprehensible. Meanwhile, inequalities determined by circumstances of birth, which an individual cannot control (e.g., sex, race, family socioeconomic status, and education level of parents) are considered unfair and objectionable.

This study also differs from the work of Contreras et al.^7^ because they have performed point estimations of IO using the index of Paes de Barros et al.^16^ Instead, this work assesses IO by comparing the entire distributions^13^ using nonparametric tests and the second-order stochastic dominance criterion^13^ ^,^ ^14^. In addition, unlike the article of Contreras et al.^7^, this study assesses IO for various circumstances, including sex, household income, education level of the mother, as well as the area and region of residence.

The main objective of this research is to test the hypothesis regarding IO in the health of Chileans. In other words, it aims to assess the presence of inequalities in health that are attributable to the characteristics beyond the control of an individual and not to differences in the effort of an individual to be healthy. In the terminology of the IO approach^18^, the research problem lies in identifying health inequalities that should be considered unfair, as they are determined by the *circumstances* and not by individual *efforts*.

The relevance of the study lies in the fact that finding evidence of IO in health will help attract greater attention from policymakers to implement compensatory measures aimed to reduce such opportunity gaps. This work contributes to the international literature in describing the application of the IO approach to health data in a developing country, given that previous studies from this perspective have been predominantly focused on developed countries^19^ ^,^ ^24^. The Chilean case is also of great academic interest since the neoliberal economic model of this country has been presented as the example to follow in Latin America, although this model has also presented deep inequalities in various areas of welfare^3^, including health.

## METHODS

The IO approach was first presented in the seminal work of Roemer^18^. This approach arises from important developments in political philosophy^6^ ^,^ ^10^ ^,^ ^17^ ^,^ ^21^ and is already strongly established in welfare economics^9^ ^,^ ^11^ ^,^ ^13^ ^,^ ^16^. According to the IO approach^18^, the welfare achievements of individuals, such as income, education, or health, depend on two factors: *circumstances* and *efforts*. The circumstances refer to the individual characteristics that are beyond individual responsibility (e.g., race, sex, parental education, parental socioeconomic status, and genetic characteristics), and they are sources of social and morally questionable inequalities. The efforts, on the other hand, depend on decisions and acts that the individual can control, and thus, they are the only sources of fair inequality. In the area of health, efforts are related to individual actions and decisions regarding personal care, for example, choosing to maintain a proper diet, exercising, and abstaining from harmful substances such alcohol, nicotine, or drugs.

To assess the presence of IO, we need to separate what part of the welfare outcome is attributable to efforts and what part is generated by circumstances for each person. However, even if there is data available on the efforts of individuals, absolute effort will not be a good mechanism to separate these two sources of inequality^18^. This is because the absolute level of effort required to achieve a certain welfare outcome also depends on the circumstances^18^. In other words, under a more favorable vector of circumstances, lower levels of effort will be required to achieve the same outcome.

In the case of health, for instance, a high-income person may require less effort to maintain a proper diet and to exercise. This is because a high purchasing power will allow a greater freedom of choice in the diet of an individual and because a higher income will provide more options to exercise (e.g., access to a gym, availability of time, or even a personal trainer or a pool at home). Therefore, for the empirical assessment of IO, the relative measures of effort are more suitable than the absolute ones.

The measure of relative effort proposed by Roemer^18^ is the percentile a person holds in the distribution of outcomes within his or her *type*. In the IO approach, *type* refers to a group of individuals who share the same vector of circumstances. We illustrate this with an example: let us suppose there are only two types of persons, corresponding to the circumstances of “being poor” and “being rich”. Then, we say that the relative effort of a poor person in percentile one of the distribution of health outcomes for the poor will be the same as that of a rich person in percentile one of the distribution of health outcomes for the rich. Thus, to evaluate the existence of IO between the two types, we should compare the conditional distributions of outcomes in relation to the circumstances of each type.

To formalize the above, we define *y* as the focal welfare variable used for the assessment of IO, between a pair of circumstances denoted as *c* and *c'*, *c* ≠ *c'*. In this article, *y* is the general health status of an adult person measured by the SRH. Following Lefranc et al.^13^, we define two statistical criteria to determine the presence of equality of opportunity. The first one, a stricter criterion, was proposed by Roemer^17^. The second criterion was proposed by Lefranc et al.^13^ The two criteria are as follows:

To achieve outcome *y*, there is a strong equality of opportunity between the circumstances *c* and *c'*, if and only if the conditional probability distributions *F* (*y|c*) and *F* (*y|c'*) are equal: *F* (*y|c*) = *F* (*y|c'*).

To achieve outcome *y*, there is a weak equality of opportunity between the circumstances *c* and *c'*, if and only if a strict second-order stochastic dominance relation (SSD) does not hold between the random outcomes conditional to such circumstances: *F* (*y|c*) ⊁_*SSD*_ *F* (*y|c'*) and *F* (*y|c'*) ⊁_*SSD*_ *F* (*y|c*).

In the above, the strict SSD, denoted as ≻_*SSD*_, is defined as follows:

*F* (*y|c*) ≻_*SSD*_ *F* (*y|c'*) ⟺ *F* (*y|c*) ⪰_*SSD*_ *F* (*y|c'*) ∧ *F* (*y|c'*) ⊁__*SSD*_ *F* (*y|c*),

such that ⪰_*SSD*_ denotes non-strict SSD and is further defined as follows:

Fyc≥SSD Fyc'⇔∫0ZFycdy≤∫0Z Fyc'dy, ∀z∈ℝ+.

In accordance with these criteria, the two null hypotheses tested are as follows: (i) a strong equality of opportunity and (ii) a weak equality of opportunity are present in the health of Chileans, over the following circumstances: education level of the mother, household income, sex, area of residence (urban or rural), and region.

To make an inference on the first hypothesis, we apply the nonparametric Wilcoxon-Mann-Whitney test, for which the null hypothesis is *F* (*y|c*) = *F* (*y|c'*). Regarding the second hypothesis, following Lefranc et al.^13^, we use the result from Shorrocks^22^, who has demonstrated that non-strict SSD is equal to the Generalized Lorenz Dominance (GLD):

*F* (*y|c*) ⪰_*SSD*_ *F* (*y|c'*) ⟺ *GL*_*F* (*y*|*c*)_(*p*) ≥ *GL*_*F* (*y*|*c'*)_(*p*), ⩝*p*,

where *GL*_*F* (*y*|*c*)_(*p*), is the Generalized Lorenz Curve (GLC) for the distribution *F* (*y|c*) evaluated in the percentile *p*. This second hypothesis is tested using graphics tools as well as applying the test of differences between the GLCs proposed by Araar^2^, under the null hypothesis *GL*_*F* (*y*|*c*)_(*p*) = *GL*_*F* (*y*|*c'*)_(*p*). The statistical analyses are performed using the Distributive Analysis Stata Package (DASP).

The data is from the 2010 ENS survey conducted by the Chilean Ministry of Health. The sampling frame for this survey is constructed from the 2002 Census of Population and Housing (Census 2002). The survey design was cross-sectional with multi-stage stratified random sampling, having national, regional, rural, and urban representation. The sample originally comprised 4,963 individuals above the age of fifteen. The questionnaire covered various aspects on health and individual characteristics. Respondents under the age of twenty were removed from the sample, assuming that early adulthood begins at the age of twenty, as in Craig^8^. Some observations with incomplete information on all the circumstances under analysis were also eliminated. The final sample comprised 4,404 individuals.

The SRH in the ENS survey is a categorical variable of the Likert-type scale that captures the general health status of the respondents, using the following five response categories: poor, fair, good, very good, and excellent. This scale follows the US model. In the European model, a scale of five categories is also used, but the response categories are different: very poor, poor, fair, good, and very good. Evidence shows the consistency between both scales^12^; moreover, the US version, which appears to be positively biased, can also generate results that are more symmetrical. In another work^1^, SRH is shown to be usually a good indicator of objective health, making it a valid indicator for an analysis of social inequalities in health.

In the ENS survey, the education level of the mother is encoded in fifteen categories from pre-school to graduate school. To simplify this variable, we recoded it into four categories defined by the last education level attained. This classification is based on the criteria established by the National Institute of Statistics of Chile (INE) for sociodemographic questionnaires. The four categories are as follows: uneducated, primary education, secondary education, and tertiary education. One limitation for this variable is that approximately 26% of the sample respondents declared that they were unaware of the education level of their mothers. Therefore, a missing data analysis was performed for this question, revealing that the distribution of SRH of the respondents who were unaware of the education level of their mothers was statistically equal to the distribution of those who reported having an uneducated mother. To verify this fact, the corresponding distributions were also compared using the nonparametric tests described above. Based on this finding, an imputation distributive criterion^15^ was applied. In other words, by the criterion of equal probability distributions, the non-response category in this question was mapped to the uneducated category. A possible explanation of the equality between these distributions could be that some individuals withheld information about the education level of their mothers because they were ashamed to admit their mothers were uneducated.

According to the 2002 census of Chile, women made up 50.7% of the Chilean population. The urban population accounted for 86.6% of the total. To simplify the regional analysis, we considered the political-administrative division of 13 regions (as of 2007), given the lower number of comparisons implied in relation to the current administrative division of 15 regions.

Regarding the circumstance of family income, we ranked household income. This ranking was supported analytically by the socioeconomic map of Chile prepared by Adimark (GfK Chile) using the data of the 2002 census. Accordingly, the population of Chile was classified into the five socioeconomic categories listed below and ordered from highest to lowest (proportions are indicated within parentheses): ABC1 (7.2%), C2 (15.4%), C3 (22.4%), D (34.8%), and E (20.3%). The observations in the sample were classified according to the household income closest to the ranges established by Adimark for the data of the 2002 census. The five groups obtained in the sample were as follows (proportions are indicated within parentheses): ABC1 (6.4%), C2 (12.8%), C3 (24.8%), D (35.0%), and E (20.9%).

## RESULTS

The two hypotheses were contrasted by pairwise comparisons of the circumstances. In other words, a total of 96 pairwise comparisons were obtained for each hypothesis: six by education level of the mother, 10 by socioeconomic stratum, 78 between regions, one by sex, and one by area of residence (i.e., urban or rural).

Table 1 presents the p-values of the Wilcoxon-Mann-Whitney test for the circumstances of “education level of the mother”, “family income”, “zone”, and “sex”. The null hypothesis of strong equality of opportunity is rejected in almost all these comparisons, even at the 1% significance level. This hypothesis is not rejected only between the circumstances of “mother with secondary education” and “mother with tertiary education”. This means that, according to the circumstance of “education level of the mother”, a strong equality of opportunity exists in health outcomes only among those individuals whose mothers have completed at least secondary school. Furthermore, the results indicate that there is no strong equality of opportunity in health in terms of sex as well as zone of residence (urban or rural).

**Table 1:** The p-values of the Wilcoxon-Mann-Whitney test, for pairwise comparison of circumstances by education level of the mother, family socioeconomic stratum, area of residence, and sex.

Variable		Education level of the mother			Socioeconomic stratum				Zone	Sex
			
		Secondary	Primary	Uneducated	C2	C3	D	E	Rural	Female
Education level of the mother										
	Tertiary	0.4905	0.0001	0.0000						
	Secondary		0.0000	0.0000						
	Primary			0.0000						
Socioeconomic stratum										
	ABC1				0.0000	0.0000	0.0000	0.0000		
	C2					0.0065	0.0000	0.0000		
	C3						0.0000	0.0000		
	D							0.0000		
Zone	Urban								0.0000	
Sex	Male									0.0000

In relation to the circumstance of “region of residence”, the results in Table 2 indicate that, in more than half of the pairwise comparisons (38 cases), the null hypothesis of a strong equality opportunity is rejected at the 5% significance level. Three regions stand out as having different opportunities from other regions: the Metropolitan Area of Santiago (Region 13) and the extreme regions of Tarapaca (Region I) in the north and Magallanes (Region 12) in the south. The likely explanation is that, on the one hand, given the strong centralization in the country, the Santiago Metropolitan Area may offer certain advantages for health such as a wider range of health services, sporting options, recreational opportunities, and educational services. Furthermore, much of the economic activity and income is concentrated in this region. On the other hand, the Tarapaca and Magallanes regions, despite being in the north and south extremes of the country, are characterized by a good quality of life and relatively high *per capita* income.

**Table 2:** The p-values of the Wilcoxon-Mann-Whitney test, for pairwise comparison of circumstances by region.

Region	2	3	4	5	6	7	8	9	10	11	12	13
1	0.0048	0.0000	0.0000	0.0410	0.0000	0.0000	0.0001	0.0000	0.0000	0.0008	0.5142	0.0432
2		0.1477	0.1000	0.5589	0.0102	0.0001	0.2029	0.0000	0.0020	0.4659	0.0014	0.1600
3			0.9942	0.0505	0.3267	0.0361	0.8434	0.0095	0.2211	0.4856	0.0000	0.0026
4				0.0453	0.2506	0.0200	0.8738	0.0035	0.1797	0.4837	0.0000	0.0014
5					0.0035	0.0000	0.0841	0.0000	0.0004	0.2288	0.0158	0.5688
6						0.2967	0.2386	0.1137	0.9939	0.0940	0.0000	0.0000
7							0.0226	0.5355	0.1999	0.0053	0.0000	0.0000
8								0.0055	0.1553	0.6156	0.0000	0.0066
9									0.0510	0.0011	0.0000	0.0000
10										0.0450	0.0000	0.0000
11											0.0003	0.0394
12												0.0177

The Figure bellow shows the comparisons of the GLC for the circumstances under analysis, with the exception of “region of residence”. When one type is dominated by the SSD criterion over another, we observe that the GLC of the dominant type goes above the GLC of the dominated type. Thus, the Figure shows that, for the circumstance of “education level of the mother”, the dominance favors the type in which the educational level of the mother is higher. For the circumstance of “socioeconomic stratum”, the dominance is observed in favor of a higher income. On the other hand, for the circumstances of “sex” and “zone of residence”, the dominance in the GLC is displayed in favor of males and residents of urban areas.

**Figure:**
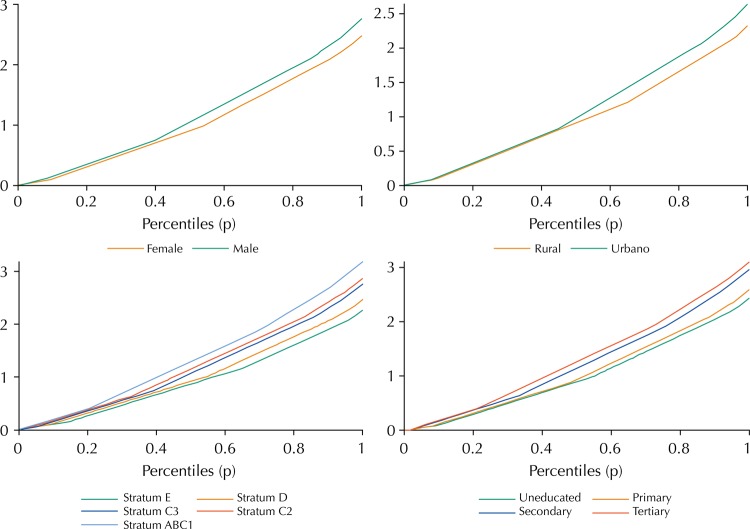
Generalized Lorenz curves according to the circumstances of the analysis.

The graphical observation of the GLC is insufficient to test the null hypothesis of weak equality of opportunity, since, even if we observe that a GLC goes over another, the difference may not be statistically significant. Therefore, to confirm the SSD, Table 3 presents the results of the Araar^2^ test to determine the statistical significance of the differences in the GLC. The results confirm almost all dominance relations of the GLC observed above in the Figure, at the 5% significance level. The only exception is the comparison of health outcomes between the circumstances of “mother with secondary education” and “mother with tertiary education”. In this comparison, the dominance of the GLC is not significant; that is, the null hypothesis of weak IO cannot be rejected in this case. This is also consistent with the finding of strong equality of opportunity between these circumstances, as mentioned above.

**Table 3:** Test results of second-order stochastic dominance (SSD) for pairwise comparison of circumstances by education level of the mother, family socioeconomic stratum, area of residence, and sex.

Variable		Education level of the mother			Socioeconomic stratum				Zone	Sex
			
		Secondary	Primary	Uneducated	C2	C3	D	E	Rural	Female
Education level of the mother										
	Tertiary	?	>	>						
	Secondary		>	>						
	Primary			>						
Socioeconomic stratum										
	ABC1				>	>	>	>		
	C2					>	>	>		
	C3						>	>		
	D							>		
Zone	Urban								>	
Sex	Male									>

The sign “>” indicates that the circumstances of the row dominate the circumstance of the column in terms of SSD. The “?” indicates that we cannot determine whether the circumstances of the row dominate that of the column in terms of SSD, and vice versa.

A comparative map of the GLC for health outcomes by region of residence is not presented here as their visual recognition is unclear (there are 13 curves). Instead, Table 4 presents the results of the Araar^2^ test. In 32 of the 78 pairwise comparisons, the null hypothesis of weak equality of opportunity between regions is rejected at the 5% significance level. Again, we can note that the extreme regions of Tarapaca and Magallanes dominate over other regions under the SSD criterion. The Santiago Metropolitan Area dominates in terms of SSD over five of the 13 regions.

**Table 4:** Test results of second-order stochastic dominance (SSD) for pairwise comparison of circumstances by region.

Region	2	3	4	5	6	7	8	9	10	11	12	13
1	?	>	?	>	>	>	>	>	>	>	?	?
2		>	?	?	>	>	?	>	?	?	<	?
3			?	?	?	?	?	?	?	?	<	<
4				?	?	>	?	>	?	?	<	?
5					?	?	?	?	?	?	<	?
6						?	?	?	?	?	<	<
7							?	?	<	?	<	<
8								?	?	?	<	?
9									<	?	<	<
10										?	<	<
11											<	?
12												>

The sign “>” indicates that the circumstances of the row dominate that of the column in terms of SSD. The “?” indicates that we cannot determine whether the circumstances of the row dominate that of the column in terms of SSD, and vice versa.

## DISCUSSION

In line with the findings of Subramanian et al.^23^, but using a different dataset and another method, this study offers new evidence that SRH and household income in Chile are strongly related. Additionally, in line with that study, we found that being a female is a disadvantage in SRH in this country. However, unlike the previous study, the SRH is not dichotomized but, instead, it includes all five categories. Therefore, the evidence presented here is more refined in its measurement of the health status. Moreover, the results of this study do not concern the inequality of outcomes of the SRH, but they reflect the IO under circumstances such as household income and sex.

The results are also consistent with the findings of Contreras et al.^7^ regarding the presence of IO in health between different regions of Chile. They have found better opportunities for good child nutrition in the Tarapaca and Magallanes regions. Similarly, this study presents evidence that these regions dominate in relation to opportunities for being healthy compared to the other regions of Chile. Meanwhile, in this study, as well as in the work of Contreras et al.^7^, the metropolitan area of Santiago emerges as one of the regions with best opportunities for good health. Naturally, we need to consider that not only the method for assessment applied but also the focal variables of health are different in these two studies.

Compared to previous studies on health inequalities in Chile, two novel findings are found in this article: Chileans enjoy better health when they have better educated mothers and if they live in urban areas.

Because of the method applied, this study offers more robust empirical evidence on social inequalities in the health of Chileans as compared to previous studies^5^ ^,^ ^7^ ^,^ ^20^ ^,^ ^25^. It is well known^14^ that the SSD criterion is more robust in establishing a hierarchical relationship between two random outcomes than in point comparisons. However, as with any research work, this study has some limitations. The method applied does not allow a differentiation between effort and luck. The development of empirical applications of the IO approach has not yet evolved enough to establish such a distinction, since it is still an emerging methodology. The nonparametric SSD criterion, although robust to test IO, does not enable us to quantify what share of inequality is explained by effort and what proportion is explained by circumstances. For future research on this topic for Chile, such quantifications could be done using parametric multivariate techniques and building counterfactual scenarios, for instance, as in the work of Trannoy et al.^24^ for France.

Regarding the contribution of this article to the international literature, its input lies in being an application of the IO approach in the context of health for a developing country. This is particularly significant given the scarce number of publications using this methodology for developed countries^19^ ^,^ ^24^.

Considering public policy implications, this work contributes by pointing out the consequences of the *compensation principle* established in the IO approach. According to this principle, inequalities derived from circumstances are unfair, and thus, they must be removed and compensated for by the focalization and reallocation of public resources.

In conclusion, this study offers robust evidence that inequalities of opportunity exist in health status in Chile depending on the education level of the mother, household income, sex, and zone and region of residence of an individual.

## References

[1] Abdulrahim S, El Asmar K (2012). Is self-rated health a valid measure to use in social inequities and health research? Evidence from the PAPFAM women’s data in six Arab countries. Int J Equity in Health.

[2] Araar A (2006). Poverty, inequality and stochastic dominance, theory and practice: illustration with Burkina Faso Surveys.

[3] Araya Rosales A, Gallardo Altamirano M (2015). El modelo chileno desde una ética de justicia y de igualdad de las oportunidades humanas. Polis (Santiago).

[4] Atkinson AB (1970). On the measurement of inequality. J Econ Theory.

[5] Castro P, Verdejo H, Garcés E, Concepción R, Sepúlveda L, Lanas F (2009). Influencia de factores socioculturales en la evolución alejada de pacientes con insuficiencia cardíaca. Rev Chil Cardiol.

[6] Cohen G (1989). On the currency of egalitarian justice. Ethics.

[7] Contreras D, Larrañaga O, Puentes E, Rau T (2012). Chile: Evolución de las oportunidades para los niños, 1990-2006. Rev Cepal.

[8] Craig GJ (2009). Desarrollo psicológico.

[9] Dunnzlaff L, Neumann D, Niehues J, Peichl A, Rodriguez JG (2011). Equality of opportunity and redistribution in Europe. Inequality of opportunity: theory and measurement.

[10] Dworkin R (1981). What is equality? Part 1: equality of welfare. Philos Public Aff.

[11] Ferreira FHG, Gignoux J (2011). The measurement of inequality of opportunity: theory and an application to Latin America. Rev Income Wealth.

[12] Jürges H, Avendano M, Mackenbach J (2008). Are different measures of self-rated health comparable? An assessment in five European countries. Eur J Epidemiol.

[13] Lefranc A, Pistolesi N, Trannoy A (2008). Inequality of opportunities vs. inequality of outcomes: are western societies all alike?. Rev Income Wealth.

[14] Levy H (1992). Stochastic dominance and expected utility: survey and analysis. Manag Sci.

[15] Little RJA, Rubin DB (2002). Statistical analysis with missing data.

[16] Paes de Barros R, Molinas Vega JR, Saavedra J (2008). Measuring inequality of opportunities for children.

[17] Rawls J (1971). A theory of justice.

[18] Roemer J (1998). Equality of opportunity.

[19] Rosa Dias P (2009). Inequality of opportunity in health: evidence from a UK cohort study. Health Econ.

[20] Sánchez-R H, Albala-B C (2004). Desigualdades en salud: mortalidad del adulto en comunas del Gran Santiago. Rev Med Chile.

[21] Sen A, McMurrin S (1980). Equality of what?. The Tanner lectures on human values.

[22] Shorrocks AAF (1983). Ranking income distributions. Economics.

[23] Subramanian SV, Delgado I, Jadue L, Vega J, Kawachi I (2003). Income inequality and health: multilevel analysis of Chilean communities. J Epidemiol Community Health.

[24] Trannoy A, Tubeuf S, Jusot F, Devaux M (2010). Inequality of opportunities in health in France: a first pass. Health Econ.

[25] Vidal Gutérrez D, Chamblas García I, Zavala Gutérrez M, Müller Gilchrist R, Rodríguez Torrres M, Chávez Montecino A (2014). Determinantes sociales en salud y estilos de vida en población adulta de Concepción, Chile. Cienc Enferm.

[26] World Health Organization (2008). Closing the gap in a generation: health equity through action on the social determinants of health: final report of the Commission on Social Determinants of Health.

